# Influence of tobacco Flower bud extract on microbial community and aroma quality during cigar leaves fermentation

**DOI:** 10.3389/fbioe.2025.1647801

**Published:** 2025-09-03

**Authors:** Lan Yao, Yule Shan, Guangyu Chen, Jiao Wang, Jingpeng Yang, Jun Yu, Chunlei Yang, Xiong Chen

**Affiliations:** ^1^ Key Laboratory of Fermentation Engineering (Ministry of Education), Cooperative Innovation Center of Industrial Fermentation (Ministry of Education & Hubei Province), College of Life and Health Science, Hubei University of Technology, Wuhan, China; ^2^ Tobacco Research Institute of Hubei Province, Wuhan, China

**Keywords:** tobacco flower bud, cigar tobacco leaves, aroma quality, metagenomics, microbial community

## Abstract

**Introduction:**

Tobacco flower buds play a crucial role in enhancing the aroma quality of cigar tobacco leaves (CTLs). By incorporating tobacco flower bud extract into the fermentation process, this study investigates its effects on microbial community dynamics and the volatile aroma compounds in CTLs, aiming to improve cigar flavor and quality during fermentation.

**Methods:**

To investigate the effects of tobacco flower bud extract on microbial communities and aroma quality during the fermentation of cigar tobacco leaves, volatile aroma components were evaluated using gas chromatography-mass spectrometry (GC-MS). The microbial community dynamics across different fermentation stages were analyzed using metagenomic sequencing.

**Results and Discussion:**

Results revealed that tobacco flower buds contain 23 characteristic aroma compounds, including β-ionone and phenylethanal. Notably, the extract induced a pronounced microbial shift, enriching *Aspergillus* in unfermented leaves and promoting *Staphylococcus* dominance (97%–98%) during fermentation. This shift facilitated carbohydrate and protein degradation, significantly reducing nicotine content (P < 0.001), increased total sugar (12.5%–18.75%) and reducing sugar levels (13.04%–27.27%), and optimized the potassium-to-chloride ratio. Aroma analysis demonstrated significant enrichment of carotenoid degradation products (farnesyl acetone, citronellal) and Maillard reaction products (5-methyl-2-furaldehyde) in the FE group, with total aroma content increasing by 11.9% compared to control (FW). Metagenomic functional analysis further indicated that the extract inhibited pathways related to harmful metabolite synthesis (47.0% reduction) and enhanced carbohydrate metabolism (30.6% increasing). This study confirms that tobacco flower bud extract reshapes microbial communities and metabolic networks by simultaneously suppressing harmful microbes and enhancing aroma, providing theoretical support for optimizing cigar fermentation and agricultural waste utilization.

## 1 Introduction

Traditional fermentation processes, representing the synergistic interaction between human practices and natural microorganism, have evolved over millennia and become deeply embedded in culinary traditions worldwide. From *lactic acid bacteria* metabolism in yogurt (Chen et al., 2017) to the saccharification and fermentation processes involving *jiuqu* (a traditional fermentation starter), microbial communities shape food flavor profiles and drive the diversity of human diets through distinct biochemical transformation pathways. This transformation process is particularly complex in the fermentation of cigar tobacco leaves, where quality development depends on both physicochemical changes and intricate microbial interactions with tobacco substrates (Yao et al., 2022b). Microorganisms colonizing cigar surfaces could degrade macromolecules such as proteins and polysaccharides, thereby facilitating aroma precursor formation and contributing to the cigar’s characteristic aroma through secondary metabolite production (Yao et al., 2023). These multi-layered biotransformation processes render microbial community succession a key factor in regulating cigar quality (Jia et al., 2023).

Traditional fermentation processes are often limited by long durations and poor controllability. Current research explores optimization strategies using exogenous additives. The use of plant extracts, microbial inoculants (Yao et al., 2022a), and other exogenous compounds can enhance fermentation efficiency by modifying the microenvironment, guiding microbial community structure, and accelerating functional metabolism (Hu et al., 2023). Notable examples include the addition of *Fangxian huangjiu* (a traditional Chinese yellow rice wine), which significantly improves microbial composition and product quality (Yao et al., 2024). The intervention of *humi* (parched rice) promotes aroma precursor formation and optimizes tobacco chemical composition (Ren et al., 2024). The combined use of plant extracts and microbial agents by Cai et al. (2024) successfully reshaped microbial communities, markedly enhancing cigar aroma richness and smoke freshness (Cai et al., 2024). These results confirm that exogenous additives influence fermentation via two mechanisms: microecological remodeling and metabolic network regulation.

**FIGURE 1:**
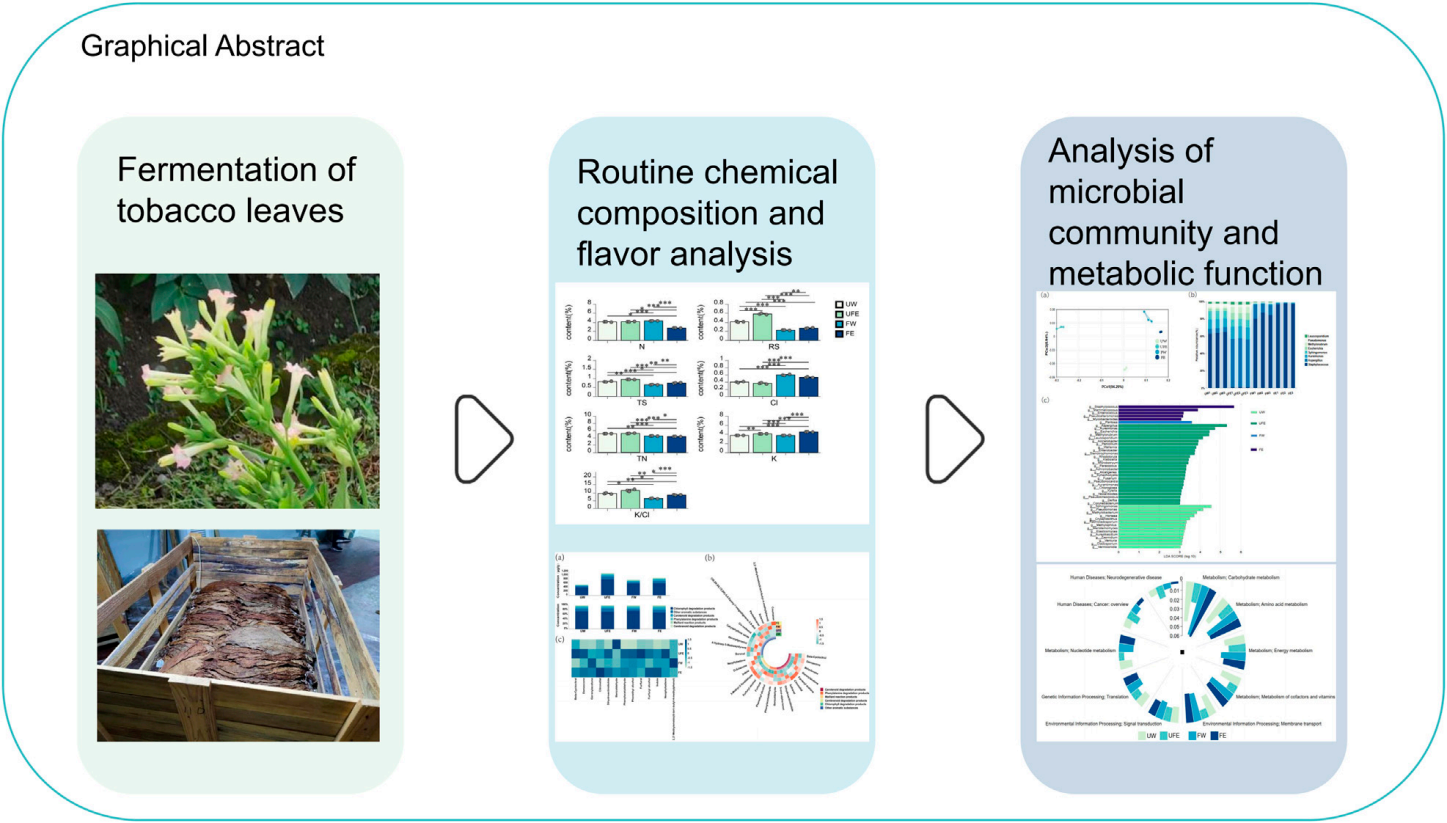
Graphical abstract.

Tobacco production generates substantial organic waste, such as tobacco flower buds, whose utilization remains limited. Improper disposal of these residues not only causes environmental pollution but also results in considerable resource waste (Shen et al., 2024). Tobacco flower buds, a byproduct of tobacco topping, are rich in aromatic compounds and nutrients, representing untapped potential (Xu et al., 2016). Although existing studies have demonstrated the positive impact of tobacco flower bud extract on the fermentation of cigar tobacco leaves (Zheng et al., 2023), critical gaps remain in understanding its underlying mechanisms. This study introduces tobacco flower bud extract into the cigar fermentation system, employing metagenomic sequencing to systematically elucidate its regulatory effects on microbial community composition, functional gene expression, and aroma compound biosynthesis. This microbial ecology-driven research strategy provides theoretical insights into improving fermentation efficiency and optimizing product flavor, while also proposing innovative approaches for utilization tobacco waste, thereby promoting circular economy principles and contributing to sustainable development goals, as graphically summarized in Figure 1.

## 2 Materials and methods

### 2.1 Experimental materials

Cigar tobacco leaves (CX-012 wrapper leaves) and tobacco flower buds were cultivated at the Enshi Prefecture Tobacco Station, Hubei Province, China, in 2023.

### 2.2 Methods

#### 2.2.1 Preparation of tobacco flower bud extract

Dried tobacco flower buds were ground into powder and mixed with water at a solid-to-liquid ratio of 1:10. The mixture was heated in a water bath at 90 °C for 2 h, cooled, and filtered to remove residues, yielding the tobacco flower bud extract.

### 2.3 Fermentation protocol

Middle-layer cigar wrapper leaves (5 kg) were evenly sprayed with water or tobacco flower bud extract. After equilibration for 4 h to restore moisture to 35%, the leaves were fermented in a constant temperature and humidity incubator at 37 °C and 80% relative humidity for 15 days. Four groups were established: FE group (fermented leaves with tobacco flower bud extract added at 15% of leaf weight), UFE group (unfermented leaves with tobacco flower bud extract), FW group (naturally fermented leaves with an equivalent volume of water), and UW group (unfermented leaves with water). For clarity and consistency, these abbreviations (FE, UFE, FW, UW) will be used throughout the manuscript.

### 2.4 Analysis of conventional chemical components in cigar tobacco leaves

Following the methods of Hu et al. (2022), chemical components were determined using a FUTURA continuous flow analyzer (Alliance Instruments, France). Total sugar and reducing sugar contents were measured according to standard YC/T 159-2019. Total nitrogen content was determined via YC/T 161-2002. Nicotine content was analyzed using YC/T 559-2018. Total potassium and chloride contents were measured following YC/T 217-2007 and YC/T 202-2006, respectively.

### 2.5 Volatile aroma compound analysis in tobacco leaves and flower buds

Dried tobacco leave/flower bud powder (10 g) was processed via simultaneous distillation extraction (SDE) using saturated NaCl solution and dichloromethane as solvents. The extract was concentrated to 2 mL, spiked with 50 µL phenethyl acetate (1.2028 mg/mL) as an internal standard, and analyzed by gas chromatography-mass spectrometry (GC-MS).

GC-MS analysis was performed using an Agilent 7890A GC coupled with a 5975C mass spectrometer (Agilent Technologies, Santa Clara, CA), equipped with an HP-5MS capillary column (30 m × 0.25 mm, 0.25 µm). Helium was used as the carrier gas at a flow rate of 1 mL/min with a split ratio of 10:1. The temperature program started at 40 °C (held for 2 min), ramped to 200 °C at 2 °C/min (held for 5 min), then increased to 280 °C at 10 °C/min. Electron impact ionization was set at 70 eV, with ion source and transfer line temperatures maintained at 230 °C and 250 °C, respectively. A solvent delay of 3 min was applied.

### 2.6 Microbial collection from tobacco leaf surfaces

Sample (30 g) was cut into fragments, immersed in 300 mL phosphate buffer, and sonicated for 30 min. The suspension was filtered through four layers of sterile gauze. The filtrate was centrifuged at 8,000 rpm for 10 min (4 °C), and the pellet was resuspended in buffer. Microbial cells were stored at −80 °C.

### 2.7 DNA extraction, library preparation, and metagenomic sequencing

Extracted DNA was amplified using primers 515F (5′-GTGCCAGCMGCCGCGGTAA-3′) and 907R (5′-CCGTCAATTCMTTTRAGTTT-3′) targeting the bacterial 16S rRNA gene. Library construction and sequencing were performed by Beijing Novogene Co., Ltd.

### 2.8 Data processing

Chromatograms were analyzed using GC-MS Solution (Agilent Technologies). Volatile compounds were identified by matching mass spectra against the NIST 14 library. Statistical analyses were conducted in Excel 2017. Significant analysis, principal coordinate analysis (PCoA), and heatmaps were generated using the CNSknowall platform (https://cnsknowall.com). Correlation analysis was performed via Bioincloud (https://www.bioincloud.tech/).

## 3 Results

### 3.1 Analysis of conventional chemical components

**FIGURE 2:**
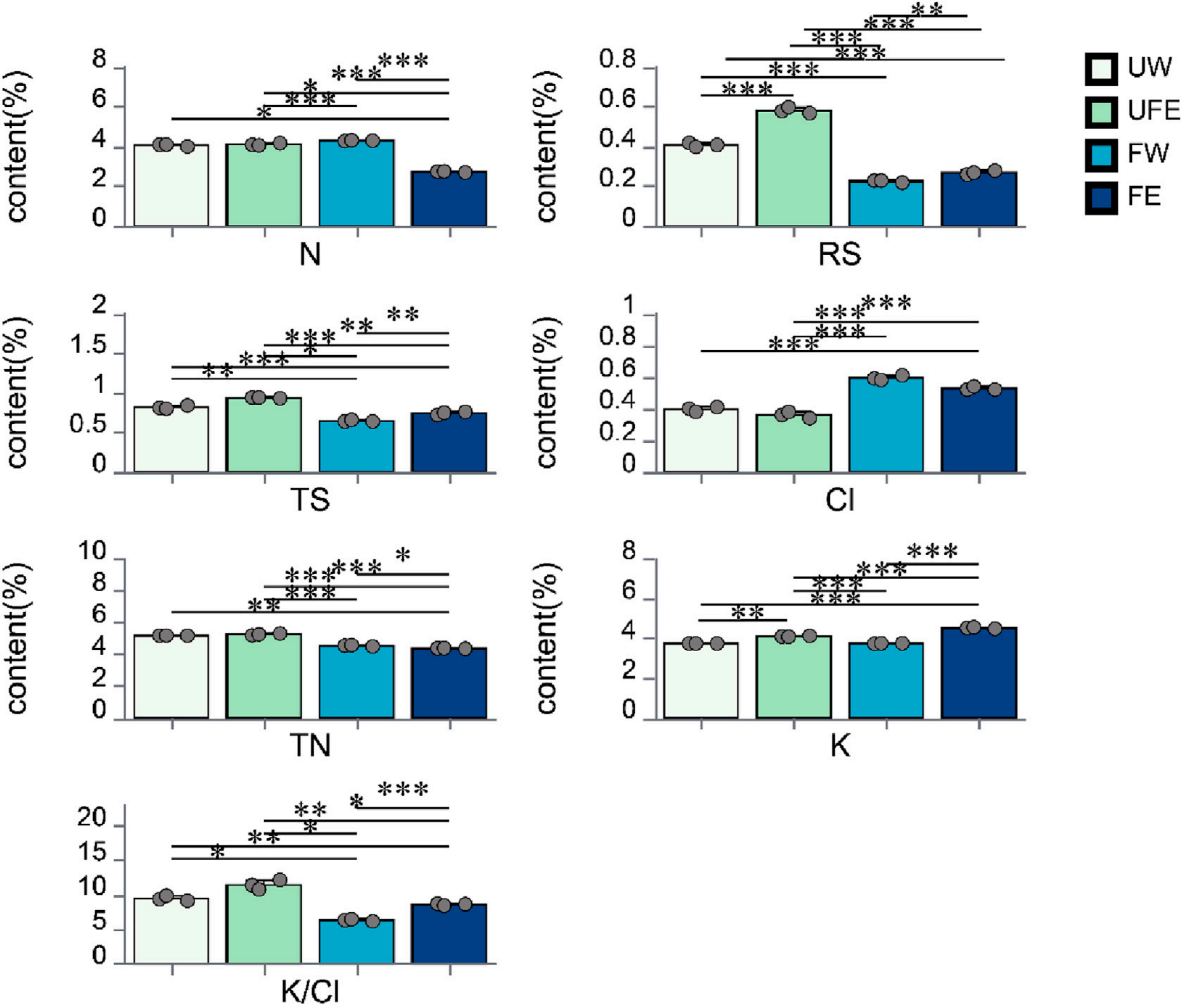
Changes in conventional chemical components during cigar tobacco leaves fermentation. N nicotine, RS reducing sugars, TS total sugars, TN total nitrogen, Cl chloride, K potassium, K/Cl potassium-to-chloride ratio.

Nicotine, reducing sugars, total sugars, total nitrogen, potassium, and chloride are key chemical components of cigar tobacco leaves, with their concentrations significantly influencing product quality. As shown in Figure 2, nicotine, reducing sugars, total sugars, total nitrogen, and potassium-to-chloride (K/Cl) ratios decreased after fermentation, partly due to microbial metabolic activity. Notably, the FE group exhibited the most pronounced reduction in nicotine content (P < 0.001), which contributes to reduced tobacco harshness and health risks. Total and reducing sugars, crucial for enhancing smoke flavor (Roemer et al., 2012), were elevated in the FE group compared to FW group. This aligns with the higher sugar content observed in the UFE versus UW group, suggesting that tobacco flower bud extract contributes sugar reserves. Previous studies have shown that tobacco flower buds are naturally rich in reducing sugars, with contents reaching up to 15.2% depending on the drying method (Wei et al., 2024).

Additionally, moderate reductions in total nitrogen during fermentation can alleviate post-smoking bitterness and astringency (Qiao et al., 2024), with the FE group showing a significant decrease in total nitrogen compared to FW group (P < 0.05).

Potassium and chloride levels critically affect cigar combustibility. A potassium-to-chloride ratio (K/Cl) of 4–10 is ideal for optimal combustion (Li et al., 2015). Post-fermentation, both FW and FE groups achieved K/Cl ratios within this range (4–10), indicating improved chemical equilibrium.

Microbial diversity and chemical components were interlinked during fermentation. Compared to FW, FE group showed reductions of 3.10%–4.13% in total nitrogen and 35.66%–37.12% in nicotine, while increasing total sugars (12.50%–18.75%), reducing sugars (13.04%–27.27%), and K/Cl ratio (30.20%–40.01%). These results confirm that tobacco flower bud extract enhances the chemical profile of fermented cigar leaves, aligning with quality optimization objectives.

### 3.2 Determination of volatile aroma compounds in tobacco flower buds

Volatile aroma compounds in tobacco flower buds were qualitatively analyzed using GC-MS. A total of 23 aroma compounds were identified (Table 1), including key components such as β-ionone, phenylethanal, 5-methylfurfural, and sclareol, which exhibited floral, fruity, and ambergris-like aromas. These compounds synergistically integrate with the inherent aroma of tobacco leaves, endowing cigars with a more complex and captivating aromatic profile (Zhang et al., 2024). As demonstrated by Bai et al. (2018), tobacco flower buds serve as an ideal substrate for Maillard reactions. When processed via enzymatic hydrolysis into tobacco flavoring agents, they could significantly enrich the aromatic complexity of tobacco leaves while enhancing the depth and subtlety of sensory attributes.

**TABLE 1:** Volatile aroma compounds in tobacco flower buds.

Cas	Aroma compounds	Cas	Aroma compounds
503-74-2	isovaleric acid	5166-53-0	5-methyl-3-hexen-2-one
4536-23-6	2-Methylhexanoic acid	14901-07-6	β-Ionone
1188-02-9	2-Methylheptanoic acid	1937-54-8	D-Solanone
105-43-1	DL-3-Methylvaleric acid	473-67-6	cis-verbenol
373-49-9	palmitoleic acid	109-20-6	geranyl isovalerate
60-33-3	linoleic acid	564-20-5	(3aR)-(+)-Sclareolide
3155-71-3	boronal	56554-41-7	glyceryl linolenate
1775-44-6	Z)-4-methylpent-2-enoic acid	22882-89-9	(2Z)-1-ethoxy-3,7-dimethylocta-2,6-diene
515-03-7	sclareol	43016-78-0	2-(dimethylamino)ethyl tetradecanoate
122-78-1	phenylacetaldehyde	67-47-0	5-hydroxymethylfurfural
628-97-7	ethyl hexadecanoate	57-10-3	Palmitic acid
7493-69-8	prop-2-enyl 2-ethylbutanoate		

### 3.3 Analysis of volatile aroma components in cigar tobacco leaves

**FIGURE 3:**
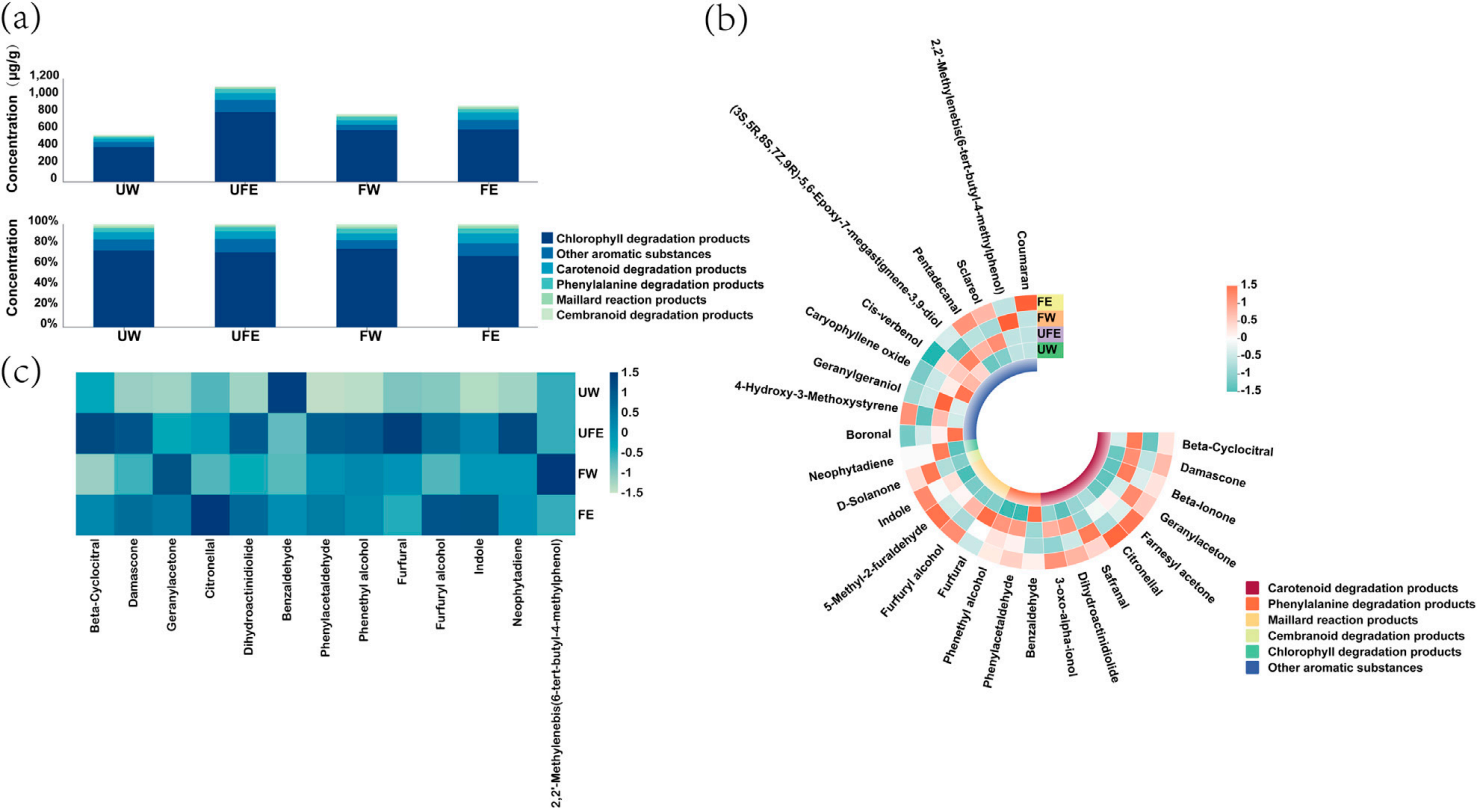
Changes in volatile aroma compounds in cigar tobacco leaves. **(a)** Bar chart of total aroma component classification, **(b)** Heatmap of intergroup aroma compound variations, **(c)** Heatmap of key aroma compounds.

As shown in Figure 3a, the addition of tobacco flower buds effectively increased the total content of volatile aroma compounds in cigar tobacco leaves. Although the total aroma content in the FE group decreased after fermentation compared to UFE (from 1,135.03 μg/g to 901.51 μg/g), it remained 11.9% higher than the FW group, likely due to microbial biotransformation during fermentation. Most aroma compounds are derived from precursors such as carotenoid degradation products, chlorophyll degradation products, and phenylalanine degradation products (Guo et al., 2024). Among them, neophytadiene, a chlorophyll degradation product, accounted for 67%–74% of the total aroma. Despite its low odor threshold, neophytadiene significantly impacts aroma quality at high concentrations, though excessive levels may contribute to grassy off-flavors in smoke (Zhang et al., 2024). Figure 3b highlights significant enrichment of carotenoid degradation products (e.g., farnesyl acetone, citronellal) and Maillard reaction products (e.g., 5-methyl-2-furaldehyde, 3-oxo-alpha-ionol, furfuryl alcohol) in the FE group. These compounds are positively correlated with tobacco aroma quality (Jia et al., 2025).

Of the 28 aroma compounds detected, 13 with olfactory activity values (OAV) > 1.0 were further analyzed using a heatmap (Figure 3c). Key contributors included β-cyclocitral, damascone, geranylacetone, citronellal, and dihydroactinidiolide. In the FE group, farnesyl acetone and citronellal -carotenoid-derived compounds imparting fruity and sweet notes (Wu et al., 2023) -as well as indole—a Maillard reaction product linked to tryptophan metabolism, contributing to floral and smooth smoke (Yang et al., 2024) -dominated the aroma profile. In contrast, the FW group was characterized by geranylacetone and 2,2′-methylenebis(6-tert-butyl-4-methylphenol). This divergence confirms that tobacco flower bud extract alters the composition of aroma-active compounds in cigar leaves.

### 3.4 Metagenomic analysis of microbial community dynamics during cigar tobacco leaves fermentation

#### 3.4.1 Microbial community diversity

Microbial community analysis was conducted on unfermented (UW), naturally fermented (FW), and tobacco flower bud-treated (FE) groups. Alpha diversity indices (ACE, Chao1, and Shannon) were used to assess species richness and evenness. All samples exhibited high microbial coverage (close to 1.0), confirming the reliability of sequencing results in representing the true distribution of surface microbiota (Table 2).

**TABLE 2:** Alpha diversity indices across experimental groups.

Index	UW	UFE	FW	FE
ace	2,342.2 ± 23.8	2,368.1 ± 22.5	1,553.2 ± 51.1	1,075.2 ± 44.4
chao1	2,365.5 ± 21.0	2,378.0 ± 36.0	1,568.3 ± 48.7	1,072.6 ± 45.7
shannon	3.58 ± 0.04	4.03 ± 0.04	1.41 ± 0.18	0.76 ± 0.06
goods_coverage	0.9999855 ± 0.0000004	0.9999935 ± 0.0000018	0.9999967 ± 0.0000004	0.9999931 ± 0.0000005

The addition of tobacco flower buds significantly affected microbial richness and diversity. As shown in Table 2, declines in ACE and Chao1 indices suggested suppressed microbial growth and reduced richness. Similarly, decreases in Shannon index suggested reduced diversity and shifts in dominant species. Although the initial microbial richness and diversity slightly increased upon flower bud addition, the overall impact was limited. After fermentation, microbial richness and diversity markedly decreased across all groups, with the FE group (treated with flower bud extract) showing the most pronounced changes.

**FIGURE 4:**
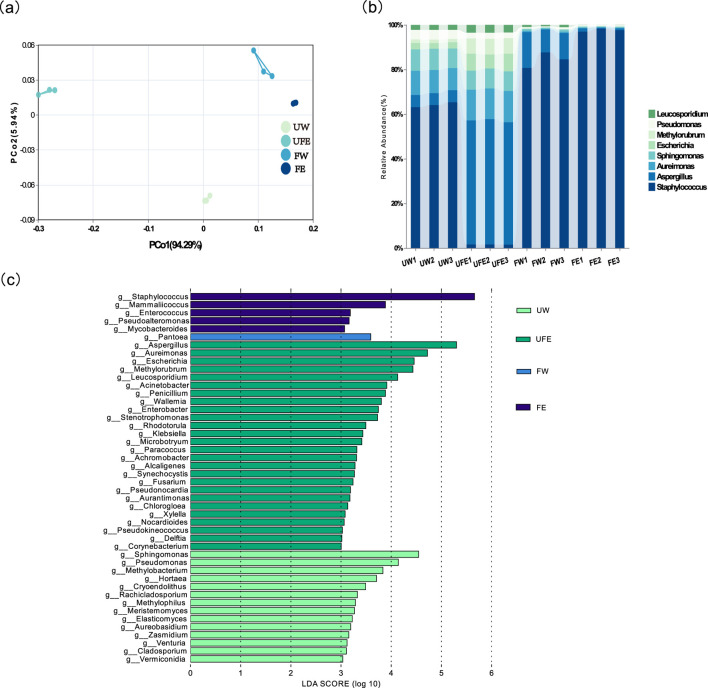
Microbial community composition of cigar tobacco leaves at the genus level. **(a)** PCoA analysis of microbial communities under different conditions; **(b)** Relative abundance bar chart of microbial communities at the genus level, **(c)** Significantly differentiated microbes across treatments at the genus level.

Principal coordinate analysis (PCoA) revealed distinct clustering of the four groups (Figure 4a). FE and FW groups clustered closer to each other but were distant from unfermented groups (UW, UFE), highlighting fermentation-driven structural shifts. Non-overlapping confidence intervals between the FE and FW groups further confirmed that tobacco flower bud extract altered bacterial community composition.

#### 3.4.2 Microbial community composition

Genus-level analysis elucidated the impact of tobacco flower buds on microbial succession (Figure 4b). In the unfermented leaves (UW group), *Staphylococcus*, *Pseudomonas*, and *Aspergillus* dominated. Upon adding flower bud extract (UFE group), *Aspergillus* replaced *Staphylococcus* as the dominant genus (63%–65% to 2%–3%), while *Escherichia* and *Methylobacterium* increased from 1%-3% to 6%–8%. After-fermentation, FW group showed enhanced dominance of *Staphylococcus* (81%–88%) and a moderate rise in *Aspergillus* (5%–10%–16%). In contrast, FE group exhibited near-exclusive dominance of *Staphylococcus* (97%–98%) with *Aspergillus* reduced to 1%.

*Staphylococcus* demonstrates a strong negative correlation with nicotine (Xue et al., 2023) and efficiently degrades carbohydrates and proteins to generate aroma compounds (Pei et al., 2025). Conversely, *Aspergillus*-a saprophytic fungus associated with tobacco mold-alters aroma profiles and compromises quality. Technical interventions (e.g., moisture control) are typically required to suppress *Aspergillus* and mitigate mold risks (Wu et al., 2024).

Genus-level microbial biomarkers with significant differences (LDA threshold >3) among different groups were identified by Linear discriminant analysis Effect Size (LEfSe) (Figure 4c), where distinct colors denote taxa enriched in specific treatments. Differentially abundant microbes spanned 46 families, with the UW group dominated by *Sphingomonas*, *Pseudomonas*, and *Methylobacterium*, UFE group by *Aspergillus*, *Aureimonas* and *Escherichia* FW group by *Pantoea*, FE group by *Staphylococcus*, *Mammaliicoccus*, *Enterococcus*, and *Pseudoalteromonas*. These results demonstrate that tobacco flower bud extract significantly alters the composition of differential microbial taxa in cigar tobacco leaves.

#### 3.4.3 Metabolic functions and shifts in cigar tobacco leaves microbiota

**FIGURE 5:**
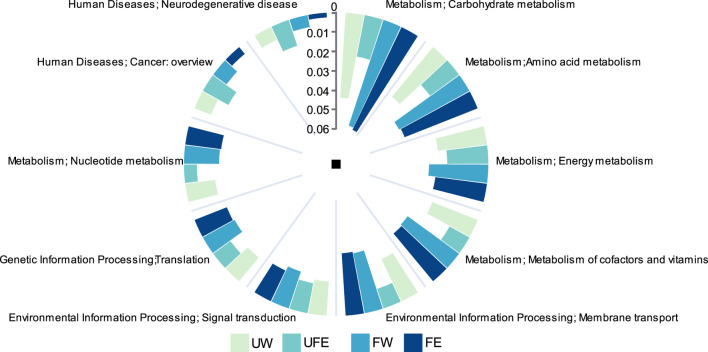
Gene abundance analysis of KEGG metabolic pathways.

Distinct microbial community structures among treatment groups led to significant alterations in metabolic pathways. KEGG pathway analysis (level 2) revealed the top five metabolic functions by relative abundance (Figure 5): carbohydrate metabolism, amino acid metabolism, energy metabolism, metabolism of cofactors and vitamins, and membrane transport, with metabolic functions predominating. Carbohydrate metabolism is critical for generating aroma precursors, as intermediates from this pathway serve as substrates for diverse compound synthesis. Similarly, amino acids participate in Maillard reactions with reducing sugars, a key source of tobacco aroma (Gao et al., 2025). Natural fermentation (FW group) significantly enhanced microbial metabolic activity, elevating carbohydrate and amino acid metabolism by 30.6% and 24.5%, respectively, compared to the UW group. The addition of tobacco flower bud extract (FE group) further optimized carbohydrate metabolism while suppressing pathways linked to human diseases, including a 47.0% reduction in carcinogen synthesis and a 61.2% decline in neurodegenerative disease-related pathways. Notably, unfermented leaves treated with flower bud extract (UFE group) exhibited transient increases in aroma precursors but showed a 50% reduction in core metabolic functions relative to the FW group, underscoring the necessity of fermentation for synergistic activation of bioactive compounds in flower buds.

## 4 Discussion

Tobacco flower buds, as a typical agricultural by-product, have traditionally been regarded as processing waste. In recent years, they have been found to be rich in natural aroma precursors and nutrients, and to possess unique potential for application in cigar tobacco leaves fermentation (Ding et al., 2024). Compared with the study by Yao et al. (2024) using *Fangxian huangjiu* (a traditional Chinese yellow rice wine), the aroma precursors of tobacco flower buds (e.g., β-ionone, phenylacetaldehyde, etc.) exhibit greater structural similarity to the tobacco leaves matrix, making them more favorable for aroma formation. Previous studies have shown that the addition of tobacco flower buds or their extracts can significantly improve tobacco quality (Nong, 2015). However, the mechanisms by which they influence microbial community dynamics and metabolic network remain unclear. Fermentation, as a key link in the cigar production process, centers on the conversion of macromolecules (e.g., proteins, sugars) into small molecules and volatile compounds under the synergistic action of microorganisms and enzymes. Traditional fermentation largely relies on natural processes but is often limited by long durations and poor controllability. In recent years, strategy involving the exogenous addition of fermentation media to enhance efficiency by modulating microbial activity have attracted gained increasing attention (Fang et al., 2024), although most studies remain limited to single-strain applications or chemical index analyses (Guo et al., 2024). In this study, we systematically revealed the multilevel regulation of microbial communities, chemical components and aroma quality of tobacco bud extracts in cigar fermentation by integrating macrogenomics and flavoromics techniques, providing a theoretical basis for the application of plant resources in fermentation engineering.

The addition of tobacco flower bud extract significantly altered the chemical profile of fermented leaves. In the FE group, nicotine content decreased markedly compared with the natural fermentation group (P < 0.001), with a 35% greater reduction than that reported by Qiao et al. (2024) for *Saccharomyces cerevisiae*-treated groups. This reduction mitigates nicotine’s harshness and associated health risks (Abrams et al., 2018), likely mediated by *Staphylococcus*-driven nicotine degradation (Pei et al., 2025). Total sugar and reducing sugar contents was increased by 12.5%–18.75% and 13.04%–27.27%, respectively. These elevated sugar levels provided sufficient substrates for Maillard reactions, helping to neutralize nicotine-induced irritation, improve smoke smoothness, and promote the generation of acids and other compounds that help stabilize smoke pH and reduce harshness. Additionally, total nitrogen content decreased markedly (P < 0.01) compared with that before fermentation (UW group). Protein and starch are macromolecules in tobacco, high levels of which are detrimental to the smoking characteristics of tobacco (Fang et al., 2024). In addition, the potassium-to-chloride (K/Cl) ratio was optimized to an optimal range of 4-10, which significantly improved the combustibility and softness of the tobacco.

GC-MS analysis results showed pronounced shifts in aroma composition. Unfermented leaves treated with flower bud extract (UFE group) exhibited the most total aroma content (1,135.03 μg/g), dominated by neophytadiene, β-ionone, phenylacetaldehyde, and 5-methyl-2-furaldehyde. Flower bud extract enriched total aroma by 106%, directly supplementing key compounds like beta-Ionone, D-Solanone, 5-methyl-2-furaldehyde, phenylacetaldehyde and sclareol (from 53.84 μg/g to 161.94 μg/g). Post-fermentation (FE group), total aroma decreased by 20.5% compared with UFE, but compositional optimization occurred: carotenoid degradation products (e.g., farnesyl acetone, citronellal) increased by 6.05%, Maillard reaction products (e.g., furfuryl alcohol) by 21.16%, and Cembranoid degradation products (e.g., D-solanone) by 26.64%, alongside a 24.6% reduction in neophytadiene to minimize grassy off-flavors. Notably, FE retained a 12.3% aroma advantage over FW, likely due to microbial metabolic shifts and accelerated chlorophyll derivative conversion during high-temperature fermentation (Wu et al., 2023). Similar to *Staphylococcus nepalensis*-enhanced carotenoid degradation (Pei et al., 2025), *Staphylococcus* dominance in FE likely drove aroma enrichment. Carotenoid derivatives (e.g., farnesylacetone’s fruity notes) and indole’s floral tones synergistically crafted the cigar’s complex aroma (Jia et al., 2025), while citronellal’s freshness enhanced aromatic complexity. Tobacco flower buds, rich in Maillard reaction precursors, have been used to develop flavor-enhancing agents (Bai et al., 2018). Cembranoid degradation products, particularly solanone-a major neutral aroma compound in tobacco-impart a fresh carrot-like sweetness that refines smoke smoothness (Yao et al., 2022b). These results underscore the necessity of synergizing tobacco flower bud extract with fermentation. The unfermented stage establishes a foundation for precursor accumulation through the flower buds’ nutrient richness, while functional microbial metabolism during fermentation achieves final aroma transformation and balance. The increased levels of key aroma compounds and optimized compositional ratios demonstrate that integrating flower bud extract with fermentation significantly enhances tobacco leaves quality.

Changes in chemical and aroma compounds were closely associated with the structure of microbial communities and their metabolic activities. Macrogenomic sequencing results showed that the introduction of the bud flower extract triggered a colony remodeling—relative abundance of *Aspergillus* surged from 5% to 63% during the unfermented stage (UFE group), while *Staphylococcus* decreased abruptly from 63% to 2%. This initial flora dominance may stem from sugars and amino acids in the extract being more compatible with the metabolic requirements of *Aspergillus*. Although rapid proliferation of *Aspergillus* may increase the risk of mold (Wu et al., 2024), its amylase activity reserves reducing sugars for subsequent fermentation (Wu et al., 2023). Its abundance was effectively suppressed to 1% by temperature and humidity regulation in the subsequent fermentation stage (FE group), achieving a balance between aroma persistence and safety. Bacterial dominance was further confirmed through LEfSe analysis, Fourteen characteristic genera were identified in the UW group (e.g., *Sphingomonas*, *Pseudomonas*, *Methylobacterium*), while 26 genera were found in the UFE group, including *Aspergillus*, *Aureimonas*, *Escherichia*, *Methylorubrum*, *Leucosporidium*. *Leucosporidium* is a psychrophilic yeast that is not adapted to the high-temperature conditions of cigar fermentation (Glushakova et al., 2021; Pan et al., 2024). Its presence in the UFE group likely originates from environmental sources or microbial residues in the flower bud extract, rather than from active fermentation. As it has no known role in aroma formation or tobacco quality improvement, *Leucosporidium* is not considered a beneficial genus in this context. After fermentation, the number of microbial genera declined from 26 species in the UFE group to 5 species in the FE group, including *Staphylococcus* and *Pseudoalteromonas*, The LDA score of *Staphylococcus* reached 4.8, indicating a shift toward microbial community specialization. *Staphylococcus* has been shown to play a dominant role in the process of tobacco fermentation, and is capable of generating aroma precursors by degrading proteins and polysaccharides, and facilitating the conversion of sugars into volatile aroma components (Hu et al., 2019). *Pseudoalteromonas* complements the metabolism of *Staphylococcus* through the secretion of lipase, amylase, and protease (Odeyemi et al., 2018) to jointly enhance the efficiency of substrate conversion. In contrast, only *Pantoea* was present in the FW group. Previous studies have shown that *Pantoea* is widely present in the brewing environment of traditional fermented foods due to its good environmental resistance. Furthermore, it has been associated with the formation of various aroma substances (Zhang et al., 2025). The predominance of *Sphingomonas* in the pre fermentation UW group suggests its potential importance at this stage. Previous studies have shown that *Sphingomonas* can be used for biodegradation of aromatic compounds (Pinyakong et al., 2003). *Aspergillus* was the dominant genus in the UFE group, and its relative abundance decreased dramatically as fermentation progressed, consistent with the results of Zhao et al. (2024).

Metabolic profiling revealed enhanced carbohydrate metabolism and membrane transport post-fermentation, underpinning substrate conversion and energy supply (Ding et al., 2023). Increased Maillard reaction intermediates (e.g., 5-Methyl-2-furaldehyde) (Abrams et al., 2018) aligned with aroma optimization. Crucially, the flower bud extract suppressed harmful metabolite pathways (e.g., carcinogens reduced by 47.0%), enhancing product safety. However, (1) *Aspergillus* suppression requires stringent humidity and temperature control, and (2) excessive *Staphylococcus* dominance may compromise diversity. Future studies should explore synergistic applications of flower bud extract with multifunctional strains (e.g., sugar-metabolizing, antimicrobial species) to refine efficiency and stability.

In conclusion, the incorporation of tobacco bud extract led to microbial community structure remodeling, metabolic network and regulation of aroma synthesis pathways, which significantly enhanced the fermentation efficiency and quality of cigar tobacco leaves. These findings offer a promising strategy for the high-value utilization of agricultural byproducts and provide a foundation for optimizing fermentation processes across diverse agricultural products.

## Data Availability

The datasets presented in this study can be found in online repositories. The names of the repository/repositories and accession number(s) can be found below: https://www.ncbi.nlm.nih.gov/, PRJNA1247888.
